# Programme of a Premium Offered by the Society of Pharmacy, of Paris

**Published:** 1850-03

**Authors:** 


Art. IV.—*Programme of a Premium offered by the Society of Pharmacy, of Paris.* Translated for this Journal from the Journal of Pharmacy, December, 1849.

It is a question, that, for a long time, has engrossed the attention of pharmaceutists, how they might be able to make a substitute for the sulphate of quinine; or, at least, so to reduce the price as to admit of its use in all cases, in which its exhibition is attended with benefit.

This question has been examined, in its different aspects, in a great number of publications: it would be use less to enter into detail, concerning the advantages attached to its solution.

The Society of Pharmacy thought that the answer to the problem might be attempted, with some chance of success; and that, in the present state of science, it might not be impossible to prepare the sulphate of quinine directly; therefore, they have resolved to make an appeal to the chemists on this subject, and hope they will receive an answer.

It would have been, but a few years since, great folly to demand the processes of chemistry to create directly, with purely inorganic elements, products generated, up to that time, solely by the influence of life.

But, in its onward march, science has shown that it already possesses a certain class of bodies, which it is able to form directly and entirely, without the living tissues, in which they are ordinarily developed.

Thus, urea, to cite an example admitted by all the world, can be produced, entirely, from its carbonate; composed of hydrogen, oxygen and nitrogen; and the time is not so far distant, we may hope, when the problem will, in all its forms, be resolved; that is, when we shall be able, an organic body being given, and its composition being known, to reproduce it, either by imitating the synthetical processes, yet unknown, by which *Nature* produces it; or by the employment of means of which chemistry, at the present day, is possessed: as in the case of urea. So far as regards the alkaloids, we are at present acquainted with a vast number, and, among them, many that are exclusively artificial products.

The modes of formation of compounds of this class, are so well known and varied, that they may easily be produced; and there is no chemist who might not be able to enjoy the satisfaction of obtaining a new alkaloid, as well as an acid, an alcohol, or an ether. Many natural organic alkalies, besides urea, have already been obtained artificially; and it is not the first time that chemists have expressed the opinion, that they will yet be able to prepare directly the alkalies of cinchona and of opium.—Messrs. Dumas, Gerhadt, Kopp, and others, have already expressed this notion.

A new and very important step has been made, towards the solution of this problem, by the remarkable work of M. Wurtz, who has made known to us a large number of artificial alkalies, derived from ammonia, and all of them capable of being expressed by one general formula $AzH^3$ ($C^m$ $H^{m-b}$) that is to say, by ammonia and carburetted hydrogen.

Each of these alkaloids constitutes, thus, one of the terms of a series, of which carburetted hydrogen, which it embraces, will be the point of departure; or, in other words, the primordial carburetted hydrogen being given, we have, by its combination with one equivalent of ammonia, an alkaloid: as we can obtain from the same radical, an alcohol, an aldehyde, an acid, or an ether, supposing it to combine with various equivalents of water and oxygen.

The principal methods by which we have thus far obtained the alkaloids are the following:

1st. The transformation of certain ammoniacal compounds—thus: Urea is obtained by the simple transformation of the cyanate of ammonia.

*Furfurine* is obtained from furfuramide.

*Amarine* is obtained from hydrobenzamide.

*Melamine* is obtained from sulphocyanuret of potassium.

2d. By the reduction of certain nitrogenized bodies by sulphuretted hydrogen or hydrosulphuret of ammonium.

*Aniline* is obtained by the reduction of nitrobenzide.

*Toluidine* is obtained by the reduction of nitrobenzoene.

*Cuminine* is obtained by the reduction of nitrocumene.

3d. By the distillation of certain organic bodies in connection with potassa. These are

*Quinoleine*, produced by the action of potassa on quinine, cinchonine, or strychnine.

*Aniline*, produced by the action of potassa on isatine.

*Conicine*, produced by the action of potassa on the unknown principle of hemlock.

*Nicotine*, produced by the action of potassa on nicotianine of tobacco.

*Valeramine*, or *amyliaque*, produced by the action of potassa on the cyanate of amyline.

4th. The dry distillation of certain products.

The distillation of coal furnishes *aniline*, known by the name of kyanole.

*Quinoleine*, known by the name of leucol.

*Pyrhol*, picoline, &c.

5th. Some alkaloids can be obtained by the desulphuration of certain oils, or sulphuretted alkaloids. Thus:

The oil of mustard desulphuretted, furnishes *sinamine* and *sinapoline*.

6th. Finally, we can, by means of natural alkalies, submitted to certain reactions, obtain a vast number of other substances possessing alkaline properties.

Such are, in particular, all the derivatives which we produce by substitution, or in replacing one or more equivalents of hydrogen in these compounds, by chlorine, bromine, iodine, etc.

For reasons, before announced, the Society of Pharmacy, of Paris, offer a reward of 4000 francs to the chemist who will discover the means of preparing artificially the sulphate of quinine; that is: without employing in the preparation, either cinchona or any other organic matter contained in quinine already formed.

In case this problem is not resolved, the reward will be bestowed on the author of the best work making known to us a new organic product, natural or artificial, having medicinal properties equal to those of quinine, and which can be placed commercially in competition with it.

The treatise must be addressed to "Le secrétaire général," of the Society, before the first of January, 1851.

Those who wish to reserve their processes, in order to preserve their ownership, should place apart, and under a sealed envelope, the descriptions they do not wish made public. On their demand, the Society will appoint one of their number to take the sole management of the experiments. He shall perform them in their presence. A process-verbal of the operations should be drawn up, before the Society separates, and will remain under seal in the hands of the secretary general of the society.

The Society will determine on the opinions of their delegate. In all cases, samples of the product must be presented to the Committee, and to the Society, to be, if it is deemed necessary, submitted to all the experiments desired.

The quantity of the sample must not be less than 250 grammes.

*Hottol, Guibourt, Bouchardat, Gaulteirde, Claubry.—Buignet et Psussy, rapporteurs.*

